# Angiogenic Potential of Human Adipose-Derived Mesenchymal Stromal Cells in Nanofibrillated Cellulose Hydrogel

**DOI:** 10.3390/biomedicines10102584

**Published:** 2022-10-15

**Authors:** Elle Koivunotko, Jasmi Snirvi, Arto Merivaara, Riina Harjumäki, Swarna Rautiainen, Minna Kelloniemi, Kirsi Kuismanen, Susanna Miettinen, Marjo Yliperttula, Raili Koivuniemi

**Affiliations:** 1 Drug Research Program, Division of Pharmaceutical Biosciences, Faculty of Pharmacy, University of Helsinki, 00790 Helsinki, Finland; 2 Department of Plastic and Reconstructive Surgery, Tampere University Hospital, 33520 Tampere, Finland; 3 Department of Obstetrics and Gynecology, Tampere University Hospital, 33520 Tampere, Finland; 4 Faculty of Medicine and Health Technologies, University of Tampere, 33520 Tampere, Finland; 5 Research, Development and Innovation Centre, Tampere University Hospital, 33520 Tampere, Finland

**Keywords:** adipose-derived mesenchymal stromal cell, endothelial differentiation, nanofibrillated cellulose hydrogel, angiogenesis, pericyte

## Abstract

Adipose-derived mesenchymal stromal cells (ASCs) hold great potential for cellular therapies by having immunomodulatory behavior and tissue regenerative properties. Due to the capability of ASCs to differentiate into endothelial cells (ECs) and other angiogenic cell types, such as pericytes, ASCs are a highly valuable source for stimulating angiogenesis. However, cellular therapies in tissue engineering have faced challenges in poor survival of the cells after transplantation, which is why a protective biomaterial scaffold is required. In this work, we studied the potential of nanofibrillated cellulose (NFC) hydrogel to be utilized as a suitable matrix for three-dimensional (3D) cell culturing of human-derived ASCs (hASCs) and studied their angiogenic properties and differentiation potential in ECs and pericytes. In addition, we tested the effect of hASC-conditioned medium and stimulation with angiopoietin-1 (Ang-1) on human umbilical vein endothelial cells (HUVECs) to induce blood vessel-type tube formation in NFC hydrogel. The hASCs were successfully 3D cell cultured in NFC hydrogel as they formed spheroids and had high cell viability with angiogenic features. Most importantly, they showed angiogenic potential by having pericyte-like characteristics when differentiated in EC medium, and their conditioned medium improved HUVEC viability and tube formation, which recalls the active paracrine properties. This study recommends NFC hydrogel for future use as an animal-free biomaterial scaffold for hASCs in therapeutic angiogenesis and other cell therapy purposes.

## 1. Introduction

Adipose-derived mesenchymal stromal cells (ASCs) are multipotent adult progenitor cells with self-renewal capacity, which are derived from stromal vascular fraction (SVF) of adipose tissue utilizing lipoaspiration [1]. As knowledge of mesenchymal stromal cells (MSCs) has increased, requirements concerning detectable surface molecules, such as CD73, CD90, and CD105, and adherence to plastic have been stated when cultured in in vitro standard conditions, which ease the detection and analyses of this heterogeneous cell population [2,3,4].

ASCs have shown increased potential for stem cell-based therapy due to their capability to interact with the immune system and to enhance cell migration and proliferation [5,6,7]. By interacting through paracrine signaling and exosome secretion, ASCs have proven immunomodulatory behavior, which would enable their allogenic or even xenogeneic utilization, and which may reduce the risk of rejection of the engraftment [8]. In preclinical and clinical settings, ASC transplantation has even been tested for the treatment of autoimmune diseases and graft-versus-host disease [4,9,10]. In addition to their immunomodulatory behavior, ASCs combined with suitable scaffolds have shown anti-apoptotic and pro-angiogenic effects together with differentiation properties that have been efficient in many regenerative tissue repair trials to treat e.g., cardiovascular diseases and bone defects [11,12]. Via release of important bioactive factors, such as growth factors and cytokines, ASCs regulate various molecular-level processes important for tissue repair, which leads to accelerated healing [13,14].

Like other mesenchymal stromal cells, ASCs have the ability to differentiate into various cell types including adipocytes, osteoblasts, and chondrocytes [1]. Especially, the ability of ASCs to differentiate into endothelial cells (ECs) and pericyte-like cells and to enhance angiogenesis as well as blood vessel formation have raised a vast amount of interest with respect to tissue engineering applications, such as in chronic wound treatment [15,16,17]. One of the most common factors promoting chronic wound formation is endothelial dysfunction causing ischemic conditions in tissue sites [18]. This encourages the utilization of EC-differentiated ASCs that have a crucial role in promoting vascular functions and secreting components that regulate important wound healing steps including cell migration and platelet activity. Alongside ECs, pericytes around the blood vessels take a crucial part in tissue regeneration processes by maintaining vascular morphogenesis and their branching [19].

The major obstacle in successful ASC transplantation is the impaired release of paracrine factors [20,21,22]. Other challenges can be faced in poor survival and low retention of cells in a target tissue due to the inflammatory environment or accumulation of cells in other tissues when administered as a single cell suspension. To overcome low cell engraftment, more emphasis is nowadays put on the development of biomaterial carriers that would support cell survival and function in addition to having tissue regenerative behavior itself. A wood-derived cellulose polymer derivate, nanofibrillated cellulose (NFC), has proven to be an efficient platform for ASC culturing in a dressing form [23] in addition to three-dimensional (3D) cell culturing and drug carrier applications [24,25]. The structure of NFC is based on highly hydrophilic cellulose fibrils with a diameter of tens of nanometers and length of several micrometers, which together with water molecules forms a homogenous NFC hydrogel. Based on the viscoelastic properties and fibril size of NFC hydrogel, it mimics the structure of the extracellular matrix (ECM). Naturally existing NFC is proven to be biocompatible and non-toxic, allowing it to be utilized in clinical applications. Our previous clinical study demonstrated NFC dressing to be suitable in skin draft donor site treatment with favorable effects on vascularization, scar thickness, and skin elasticity [26]. By having these properties, NFC hydrogel could be used as a protective carrier and suitable matrix for differentiation of ASCs.

In this work, our aim was to study the angiogenic properties and cell characteristics of donor-derived human adipose-derived mesenchymal stromal cells (hASCs) and to induce their differentiation towards a vascular lineage, i.e., endothelial cells or pericyte-like cells, in NFC-hydrogel-based 3D culture. We characterized hASCs for their expression of ASC, pericyte, or EC-specific markers and angiogenic factors. Additionally, we created a 3D angiogenesis model of human umbilical vein endothelial cells (HUVECs) in NFC hydrogel to test the effects of a conditioned medium of 2D- and 3D-cultured hASCs on their viability and endothelial cell tube formation. NFC hydrogel holds great potential to be utilized as a transplantation material for cells at the site of action to improve their performance for accelerated tissue regeneration.

## 2. Materials and Methods

### 2.1. Cells

Donor-derived hASCs were isolated from adipose tissue samples acquired from surgical procedures at the Department of Plastic Surgery, Tampere University Hospital, and HUVECs from umbilical cord acquired from scheduled Cesarean sections at the Department of Obstetrics and Gynecology, Tampere University Hospital. The study was carried out in accordance with the Ethics Committee of Pirkanmaa Hospital District, Tampere, Finland (R15161 and R13019), and written informed consent was provided by the patients. HUVECs from PromoCell, Heidelberg, Germany (Cat. #C-12203) were used to prepare control samples for qRT-PCR and Western blotting experiments. Human adipose-derived stromal cells from Lonza, Basel, Switzerland (Cat. #PT-5006) were used to collect 2D and 3D hASC conditioned medium for HUVEC 3D cell assay at passages four to seven.

### 2.2. Human ASC Isolation and Characterization

Human ASCs were isolated and characterized as described previously by Kyllönen et al. [27]. The cells were obtained from subcutaneous adipose tissue of nine donors (eight females, one male; mean age 48.3 ± 11.3) using Dulbecco’s Modified Eagle Medium/Ham’s Nutrient Mixture F-12 (DMEM/F12, Cat. 21331-020; Gibco, Thermo Fisher Scientific, Waltham, MA, USA) supplemented with 5% (*v*/*v*) human serum (Cat. #C15-021; PAA Laboratories (Pasching, Austria), 1% (*v*/*v*) penicillin/streptomycin (PS; Cat. #DE17-602E; Lonza (Cologne, Germany) and 1% (*v*/*v*) L-alanyl-L-glutamine (GlutaMAX, Cat. #35050-038; Gibco, Thermo Fisher Scientific). Cells were characterized by their ability to differentiate towards adipocyte and osteogenic lineages using Oil red O (Cat. #O0625; Sigma-Aldrich, Burlington, MA, USA) and Alizarin red S (Cat. #A5533; Sigma-Aldrich) stainings, respectively, and by cell surface marker expression using flow cytometry as described previously by Vuornos et al. [28] using antibodies listed in Table S1. Characterizations were done at passages two to three. Results indicated a mesenchymal origin of the isolated hASCs (Table S2; Figure S1).

### 2.3. HUVEC Isolation and Characterization

Donor-derived HUVECs were extracted from the umbilical cord according to Hamilton et al. [29]. Briefly, the cord was separated from the placenta; the umbilical vein was cannulated with a 20 G needle, and the needle was secured by clamping the cord over the needle with a clamp. The vein was perfused with Dulbecco’s phosphate-buffered saline without calcium and magnesium (DPBS, Cat. #17-512F; Lonza) to wash out blood, and then, the opposing end of the umbilical vein was clamped. Subsequently, the vein was infused with collagenase II (Cat. #C6885; Sigma-Aldrich). The umbilical cord was incubated in a water bath at +37 °C for 15 min. After incubation, the collagenase solution containing HUVECs was flushed from the cord into a 50 mL polypropylene tube. The cells were centrifuged at 1200 rpm for 6 min and resuspended in EGM-2 BulletKit (Cat. #CC-3162; Lonza) medium supplemented with 2% human serum (Cat. #S4190-100, BioWest, Nuaillé, France) and seeded. The HUVECs were cultured at +37 °C in 5% CO_2_, and the medium was changed twice per week. To verify the endothelial phenotype of HUVECs, surface marker expression was characterized by flow cytometry (FACSAria; BD Biosciences, Erembodegem, Belgium) as described previously [30].

### 2.4. Cell Culturing

Donor ASCs were used for experiments between passages three to six and cells isolated from individual donors served as replicates (n numbers refer to the number of donors, i.e., the number of repeats of separate experiments with cells from different donors). Human ASCs were cultured in MEM α GlutaMAX™ Supplement medium (MEM-α, Cat. #32571036; Gibco, UK) supplemented with 5% (*v*/*v*) of human serum (Cat. #H4522; Sigma-Aldrich).

HUVECs were used for experiments between passages four to six. Cells were cultured in EBM-2 Basal Medium (Cat. #CC-3156; Lonza) supplemented with EGM-2 Endothelial SingleQuots Kit (Cat. #CC-4176; Lonza) or in Endothelial Cell Growth Basal Medium 2 (Cat. #C-22216; PromoCell) supplemented with Growth Medium 2 Supplement Mix (Cat. #C-39216; PromoCell).

Cell culturing was performed at +37 °C in a humified cell culture incubator with 5% CO2. Cell morphology and growth were evaluated with Leica DM IL LED microscope (Leica Microsystems, Wetzlar, Germany).

### 2.5. 3D Culturing of hASCs

To evaluate optimal 3D cell culture conditions, human ASCs were cultured in different NFC hydrogel (GrowDex^®^, Cat. #100103002; UPM Biomedicals, Lappeenranta, Finland) fiber concentrations (0.125%; 0.25%; 0.5%). Based on the rheological measurements, further studies were continued with 0.125% NFC fiber concentration (Supplementary Information). Cell seeding to NFC hydrogel was performed as described previously by Bhattacharya et al. [25]. Briefly, the cells were detached from culture surface and seeded with 20,000 (20 k), 50 k, 70 k, or 100 k cells per 100 µL density in NFC hydrogel diluted in hASC growth medium, on low adhesion 96-well inertGrade BRAND plates^®^ (Cat. #BR781902; Merck, Darmstadt, Germany). After 15–20 min, 120 µL of the growth media was pipetted on top of the hydrogel. Medium change for 3D cultures was performed 2–3 times per week after centrifuging the well plate at 150× *g* for 7 min. Spheroid diameters were measured using LAS EZ 3.4.0 software (Leica Microsystems). Conditioned media (CM) from hASC 2D cultures or 3D cultures on day 1 were collected and stored at −80 °C and used for stimulation of donor HUVECs.

### 2.6. Endothelial Differentiation of hASCs

For endothelial differentiation, hASCs were seeded at 100 k cell density per 100 µL of 0.125% NFC hydrogel diluted in endothelial cell growth medium (EBM-2 Basal Medium supplemented with EGM-2 Endothelial SingleQuots Kit, Lonza), on low adhesion 96-well inertGrade BRAND plates^®^, and 120 µL of the media was pipetted on top of the hydrogel. Cultures were kept for 21 days with a medium change twice a week. Some of the cultures were stimulated with 100 ng/mL of recombinant human Angiopoietin-1 (Ang-1) (Cat. #130–06; Peprotech, London, UK) upon seeding and during medium changes.

### 2.7. 3D Angiogenesis Model of HUVECs

Donor-derived HUVECs were 3D-cultured in 0.5% NFC hydrogel utilizing a similar protocol as described above for hASCs. Cells were seeded with 30 k cells per 50 µL density in NFC hydrogel diluted in medium with and without 450 ng/mL of Ang-1 or with 2D or 3D hASC CM. Medium change was performed on the third day after seeding during one-week culturing. Staining was based on the protocol by Mimler et al. [31]. Briefly, on day 7, fixing protocol was implemented on HUVECs with 100 µL/well 4% PFA with 0.8% Triton X-100, 5 mM EDTA (Cat. # E6758; Sigma-Aldrich), and 1 mM MgCl_2_ (Cat. #63063; Sigma-Aldrich) for 20 min at room temperature, after which the cells were washed three times with PBS. After that, cells were blocked with 50 µL/well with 3% BSA and 0.3 M glycine in PBS-T for 1 h at room temperature. Primary antibody staining (2 h at room temperature) was implemented with anti-rabbit CD31/platelet endothelial cell adhesion molecule (PECAM-1) (1:200; Cat. #NB100-2284; Novus Biologicals, Abingdon, UK), and anti-rabbit von Willebrand Factor (vWF) (1:200; Novus Biologicals) antibodies in 3% BSA/PBS-T. The next day, cells were washed three times with PBS-T and incubated with a secondary antibody, donkey anti-rabbit Alexa 594 1:500 (Cat. #A21207; Invitrogen) in 5% BSA/PBS-T for 2 h at room temperature. After washing with PBS-T and 0.1 M Tris-HCl pH 7.4, nuclei stain Hoechst 4 µg/mL (Cat. #B2261; Sigma-Aldrich) in 0.1 M Tris-HCl pH 7.4 was added for 2 h. After final washing with PBS-T, cells were stored in PBS. Cells were imaged with Leica TCS SP8 STED 3X CW 3D confocal microscopy (Leica microsystems, Wetzlar, Germany) and the images were processed with Leica Application Suite X (Leica microsystems) and Fiji ImageJ 1.53 software.

### 2.8. Cell Viability

Cell viability was evaluated as mitochondrial metabolic activity using alamarBlue™ Cell Viability Reagent (Cat. #DAL1025; Invitrogen, Thermo Fisher Scientific). AlamarBlue™ solution was diluted with growth media to a final volume of 10% (*v*/*v*) and 100 µL of the solution was added to hASCs embedded in NFC hydrogel and incubated for three hours at +37 °C. After the incubation, the solution was collected from cells as during normal media change and spun down for 15 s and 80 µL of the supernatant was transferred to a black 96-well plate (Nunc^®^ MicroWell 96 optical bottom plates, Cat. #P8866-30EA; Sigma-Aldrich) for fluorescence measurement using Varioskan LUX and SkanIt RE-program 6.1 (Thermo Fisher Scientific) at excitation of 560 nm and emission of 590 nm. The fluorescence signal was normalized to the signal from 2D-cultured control cells seeded as 30,000 cells/cm^2^ on a 96-well plate (Cat. #83.3924, Sarstedt, Nümbrecht, Germany) and blank control samples containing hydrogel without cells, except in experiments presented in Supplemental information, where the fluorescence signal was normalized only to the signal from blank control samples.

For the evaluation of cell viability of ECs, donor HUVECs were seeded on a 96-well plate (Cat. #83.3924, Sarstedt, Germany) coated with EmbryoMax^®^ 0.1% gelatin solution (Cat. #ES-006; Merck, Darmstadt, Germany) with 5000 cells per 100 µL of growth medium per well and incubated for 24 h, after which the medium was changed to 50% 2D- or 3D- cultured hASC CM in EC growth medium. Control cells were incubated with EC growth medium. After 48 h of exposure time to hASC CM, cell viability was determined as described above by adding 100 µL of the 10% (*v*/*v*) alamarBlue™ solution on the cells.

### 2.9. Immunocytochemistry of hASC Spheroids

Human ASC spheroids cultured in NFC hydrogel were recovered using enzymatic digestion of the cellulose with cellulase mixture (GrowDaze^®^, Cat. #900102001; UPM Biomedicals) for 20–24 h according to the manufacturer’s instructions. Spheroids were collected into a low-bind Eppendorf tube and fixed with 4% paraformaldehyde (PFA) for 20 min in rotation. After that, cells were washed three times with 0.1% (*v*/*v*) Tween 20 detergent (Cat. #P9416; Sigma-Aldrich) in phosphate-buffered saline (PBS). Spheroids were then permeabilized in 0.1% (*v*/*v*) Triton-X-100 (Cat. #X100; Sigma-Aldrich) in PBS (PBS-T), centrifuged at 300× *g* for 5 min and transferred to a microscope glass in a small volume of PBS. After 20 min, the cells were blocked with PBS-T containing 3% (*m*/*v*) bovine serum albumin (BSA, Cat. #P6154; Biowest, France) and 0.3 M glycine (Cat. #33226; Sigma-Aldrich) for one hour at room temperature. After blocking, the cells were incubated with anti-mouse Vimentin antibody (1:50, Cat. #sc-6260; Santa Cruz Biotechnology, Santa Cruz, CA, USA) or conjugated Phalloidin Alexa 488 (1:40, Cat. #A12379; Thermo Fisher Scientific) in PBS-T containing 3% (*m*/*v*) BSA overnight at +4 °C. On the following day, cells incubated with an unconjugated antibody were washed three times with PBS-T before adding Alexa Fluor 488 goat anti-mouse IgG (1:500, Cat. #A11001; Life Technologies, Carlsbad, CA, USA) in PBS-T containing 5% (*m*/*v*) BSA. Subsequently, all the cells were washed three times with PBS-T and mounted with ProLong Diamond Antifade Mountant with DAPI (Cat. #P36966; Invitrogen) and covered with cover glass (Cat. #15747592; Menzel-Gläser, Braunschweig, Germany). Negative control samples were stained with the secondary antibodies only. Samples were imaged with Aurox Clarity Laser Free Confocal HS (Aurox, Oxford, UK) widefield microscopy and analyzed with Fiji ImageJ 1.51 software.

### 2.10. Quantitative Real-Time PCR (qRT-PCR)

For RNA extraction, hASC spheroids cultured in NFC hydrogel were recovered using cellulase enzyme mixture as described above. Human ASCs, or HUVECs cultured on tissue culture plastic coated with EmbryoMax^®^ 0.1% gelatin solution (Cat. #ES-006; Merck Millipore, Germany), were washed twice with ice-cold 1x DPBS (Cat. #14200-067; Gibco) before lysis. Total RNA was extracted using an RNeasy^®^ Mini Kit (Cat. #74104; Qiagen, Hilden, Germany) and cDNA was prepared from total RNA using a High-Capacity RNA-to-cDNA Kit (Cat. #4387406; Applied Biosystems, Thermo Fisher Scientific according to the manufacturer’s instructions. Fast SYBR^®^ Green Master Mix (Cat. #4385612; Applied Biosystems, Thermo Fisher Scientific) was applied to prepare qRT-PCR reactions using a total volume of 20 µL, and 2 µL of cDNA as a template. QRT-PCR was performed on a StepOnePlus Real-Time PCR System with StepOne Software v2.3 (Applied Biosystems, Thermo Fisher Scientific). Reactions were run in triplicate including a non-template control (water) and a non-amplification control (no SYBR^®^ Green). β-2-Microglobulin (β-2-m) was used as a housekeeping gene. The following conditions were used for qRT-PCR: an initial activation and denaturation step of 95 °C for 30 s, 40 amplification cycles consisting of 95 °C for 5 s, 60 °C for 15 s, and 72 °C for 10 s, and final elongation at 72 °C for 1 min. The relative quantification of gene expression levels in comparison with the housekeeping gene was analyzed using the relative standard curve method, with 2-fold serial dilutions of a control sample prepared for the standard curve. The used primer sequences are listed in Table 1.

### 2.11. Enzyme-Linked Immunosorbent Assay (ELISA)

For quantification of VEGF secretion, 2D-cultured hASCs seeded as 300,000 cells/cm^2^, and 3D-cultured hASCs were stimulated overnight with 10 µg/mL of lipopolysaccharide (LPS) from *Escherichia coli* (Cat. # L6529; Sigma Aldrich), and conditioned medium (CM) was collected. Measurements were performed in triplicate by Human VEGF DuoSet ELISA (Cat. #DY293B, R&D Systems, Minneapolis, MN, USA) according to the manufacturer’s instructions using Clariostar and the Mars v.3.32 software (BMG Labtech, Ortenberg, Germany). The optimal density was detected at a wavelength of 450 nm with wavelength correction measurement at 540 nm. Values from duplicate measurements were normalized to the background signal and the extinction obtained from standard curves.

### 2.12. Western Blotting

Cells were washed twice with ice-cold DPBS and homogenized in ice-cold Pierce RIPA lysis buffer (Cat. #89900; Thermo Scientific, Finland) supplemented with cOmplete™ Protease Inhibitor Cocktail (Cat. #11697498001; Roche, Switzerland) and PhosSTOP™ phosphatase inhibitor (Cat. #4906845001; Roche). Before lysis, 3D-cultured hASCs were recovered using cellulase enzyme mixture as described above. Protein concentrations were determined using the Pierce BCA Protein Assay Kit (Cat. #23225; Thermo Scientific). Equal amounts of protein (15 µg) were separated by SDS-PAGE and transferred onto a nitrocellulose membrane using Trans-Blot Turbo Mini Nitrocellulose Transfer Pack (Cat. #1704158; Bio-Rad Laboratories, Finland) and Trans-Blot Turbo Transfer System (BioRad) in 20 mM Tris-HCl, pH 7.4, 150 mM NaCl, 0.1% Tween 20 (TBS-T), and using 5% non-fat milk for 1 h at room temperature, followed by incubation at +4 °C overnight with following primary antibodies: anti-rabbit ACTA2/alpha-smooth muscle actin (α-SMA) (1:1000, Cat. #LS-B16663; LifeSpan Biosciences Inc, Seattle, WA, USA), anti-rabbit CD31/PECAM-1 (1:500), anti-mouse NG2/MCSP (1:200, Cat. #NBP2-80873; Novus Biologicals), and anti-rabbit β-Actin (1:300, Cat. #AHP2417; Bio-Rad Laboratories). Membranes were washed and exposed to peroxidase conjugated goat anti-rabbit IgG (1:2000, Cat. #A16096; Invitrogen) or goat anti-mouse IgG (1:500, Cat. #A10551; Life Technologies, Carlsbad, CA, USA) secondary antibodies for 1 h at room temperature and detected with Pierce ECL Western Blotting Substrate (Cat. #32209; Thermo Scientific) using ChemiDoc MP Imager and Imagelab software (Bio-Rad Laboratories). Some of the membranes were stripped with 0.1 M glycine for 20 min at room temperature and reprobed using anti-mouse β-actin (1:5000, Cat. #A5441; Sigma-Aldrich) antibody followed by incubation with secondary antibody. Relative amounts of proteins were quantified using ImageJ 1.50i software.

### 2.13. Statistical Analysis

Data are expressed as mean ± standard deviation (SD). Statistical significance between two groups was analyzed using Student’s t-test and differences between three or more groups using one-way ANOVA, followed by a Tukey HSD post hoc test. Values of *p* < 0.05 were considered statistically significant.

## 3. Results

### 3.1. Culturing of hASCs in NFC Hydrogel

**Figure 1:**
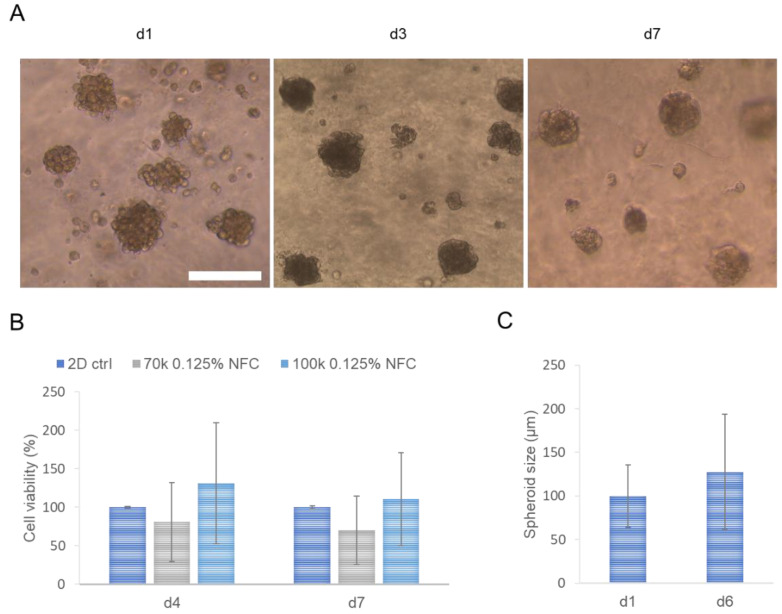
Culturing of human adipose-derived mesenchymal stromal cells (hASCs) in nanofibrillated cellulose (NFC) hydrogel. (**A**) Light microscopy images of hASC spheroids in NFC hydrogel on day 1 (d1), d3, and d7. Scale bar, 200 µm. (**B**) Cell viability of hASCs with cell densities 70,000 (70 k) and 100 k per well cultured in 0.125% NFC hydrogel compared with control two-dimensional (2D) culture on d4 and d7 (*n* = 3; 3 technical replicates). (**C**) Measured diameters of hASC spheroids (100 k) in NFC hydrogel on d1 and d6 (*n* = 2–5; >5 technical replicates). Ctrl, control.

In the beginning, cell viability was evaluated with various hASC cell densities (20 k; 50 k; 100 k) embedded in NFC hydrogel with different fiber concentrations (0.125%; 0.25%; 0.5%). Similar cell viability levels were observed regardless of the hydrogel concentration (Figure S2). However, 100 k cell density showed improved cell viability compared with other cell densities. We then measured Young’s modulus of various NFC hydrogel concentrations to determine optimal fiber concentration with respect to the native stiffness of adipose tissue [32], the origin of hASCs, which is considered as soft tissue. We chose to use fiber concentration of 0.125%, which has a low stiffness of 2 Pa (Figure S3A). In subsequent rheological measurements, we observed that the loss modulus (G”) of 0.125% NFC hydrogel was higher compared with the storage modulus (G’) (Figure S3B). In 3D culture, hASCs formed spheroid structures with grape-like or round morphology during 7-day culturing (Figure 1A; [33]). Cell culturing of hASCs with 70 k and 100 k cell density in 0.125% NFC hydrogel showed the highest cell viability with 100 k cell density during one week of culturing, which was slightly higher when compared to that of 2D culture although it did not reach statistical significance (Figure 1B). The measured spheroid sizes were below 200 µm in diameter (Figure 1C).

### 3.2. Characteristics of hASCs Were Mostly Retained in NFC Hydrogel 3D Culture

**Figure 2:**
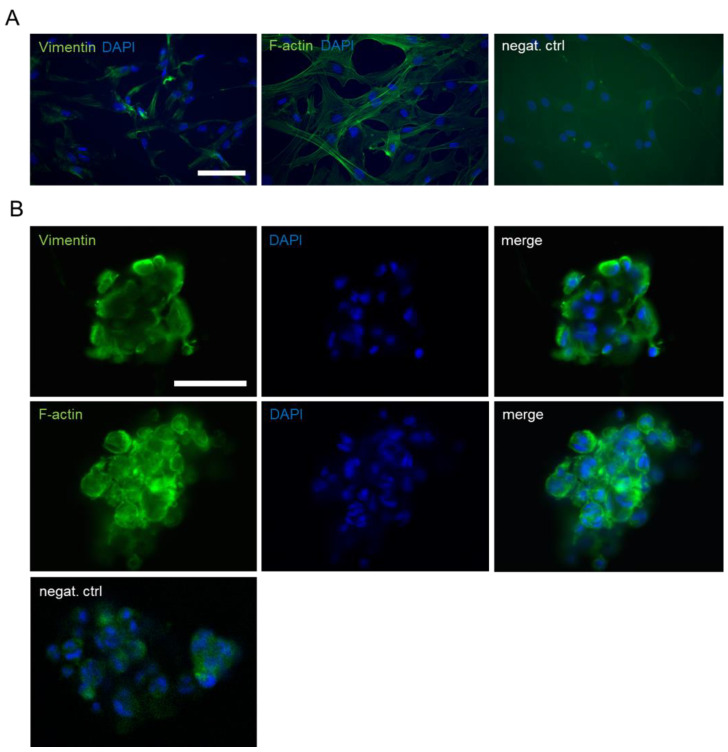
Immunocytochemistry of hASC cultures. (**A**) Expression of Vimentin and filamentous actin (F-actin) in 2D-cultured hASCs. Scale bar, 100 µm. (**B**) Vimentin and F-actin-expressing hASC spheroids cultured in NFC hydrogel with 100 k cell density on d7. Scale bar, 50 µm. Nuclei were stained with DAPI.

We further characterized hASCs cultured with 100 k cell density in 0.125% NFC hydrogel using immunocytochemistry and quantitative real-time PCR (qRT-PCR). The staining of vimentin, a mesenchymal marker, showed positive expression in hASC spheroids similar to 2D-cultured cells (Figure 2A,B). Filamentous actin (F-actin) staining confirmed the grape-like morphology of hASC spheroids (Figure 2B).

**Figure 3:**
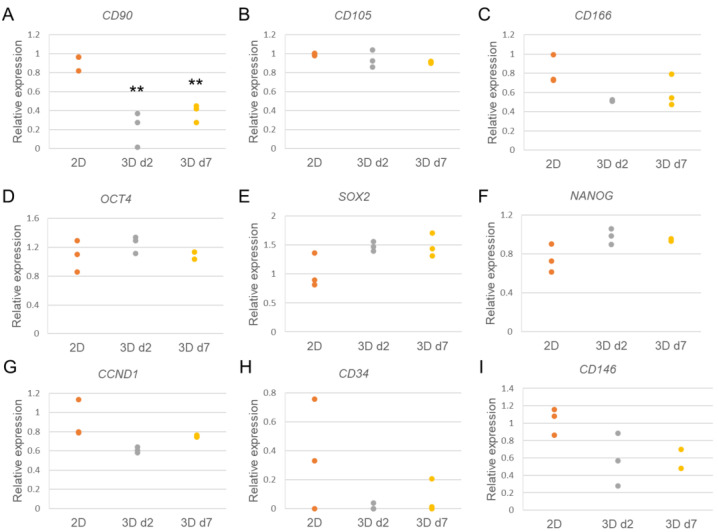
Quantitative real-time PCR (qRT-PCR) of specific cell markers in hASCs cultured in NFC hydrogel with 100 k cell density on d2 and d7 compared with 2D-cultured control cells. (**A**–**C**) Relative expression of ASC-specific cell surface markers *CD90* (**A**), *CD105* (**B**), and *CD166* (**C**). (**D**–**F**) Relative expression of pluripotency markers *OCT4* (**D**), *SOX2* (**E**), and *NANOG* (**F**). (**G**) Relative expression of a cell cycle activator *CCND1*. (**H**,**I**) Relative expression of endothelial-specific cell surface markers *CD34* (**H**) and *CD146* (**I**). ** *p* < 0.01 vs. 2D. N = 3 (3 technical replicates), except for 3D d7 samples on *OCT4*, *NANOG,* and *CD146* qRT-PCR *n* = 2 (3 technical replicates).

Surprisingly, analysis of hASC gene expression revealed decreased expression of a mesenchymal specific cell surface antigen *CD90* in 3D-cultured hASCs compared with 2D control cells (Figure 3A). Other cell surface antigens specific for hASCs, including *CD105* and *CD166,* or the pluripotency markers *OCT4*, *SOX2,* and *NANOG*, on the other hand, did not show a significant change (Figure 3B–F). A positive cell cycle regulator *CCND1* [33], as well as endothelial lineage-specific cell surface antigens *CD34* and *CD146,* showed lower values but no statistical significance in 3D-cultured hASCs compared with 2D-cultured cells (Figure 3G–I). *CD34* expression, however, showed especially high variability in 2D-cultured control cells between different donors, and very low expression levels in 3D-cultured hASCs (Figure 3H). Similar expression profiles to these were also obtained when 70 k cell density was applied in 3D cultures (Figure S4). The results indicate that hASC characteristics, including mesenchymal properties, stemness, proliferation capacity, and endothelial cell potential were mostly sustained, except of *CD90* expression, in NFC hydrogel 3D culture.

### 3.3. Differentiation of hASCs in Endothelial Cell Growth Medium

**Figure 4:**
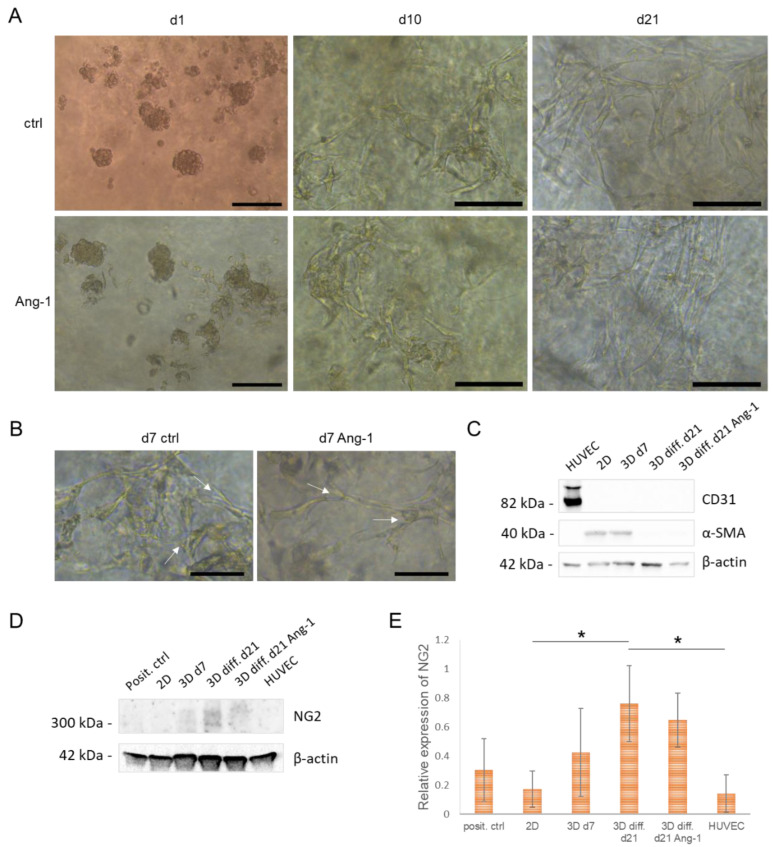
Differentiation of hASCs towards endothelial cells (ECs) in NFC hydrogel. (**A**) Differentiation of hASCs in 0.125% NFC hydrogel in endothelial growth medium for 21 days. Scale bars, 100 µm (d1) and 50 µm (d10, d21). (**B**) Differentiated cells in 0.125% NFC hydrogel on d7 showing branching of cellular structures (arrows). Scale bars, 25 µm. (**C**) Western blot showing expression of endothelial specific marker CD31 and alpha-smooth muscle actin (α-SMA) in hASCs vs. human umbilical vein endothelial cells (HUVEC) (*n* = 3, 1 technical replicate). (**D**) Western blot of pericyte marker NG2 in hASCs and HUVECs. (**E**) Quantification of NG2 expression (*n* = 3, 1 technical replicate). * *p* < 0.05. Ang-1, angiopoietin-1; diff., differentiated cells.

With respect to decreased expression level of *CD90*, we hypothesized that hASCs may be prone to differentiation in NFC hydrogel. Therefore, we tested the capability of hASCs to differentiate towards ECs or perivascular cells, which are important in order to support angiogenesis. Cells were seeded in EC growth medium in 0.125% NFC hydrogel and cultured for up to 21 days to ensure differentiation and possible interconnected tube formation [15]. Some of the cultures were stimulated with 100 ng/mL of angiopoietin 1 (Ang-1), which can induce the formation of functional microvascular networks from human mesenchymal stromal cells [34]. During the culturing, hASCs changed their appearance from spheroids to elongated capillary-like structures (Figure 4A). With Ang-1 stimulation, cultures showed a faster change in cell morphology, which was apparent already after 1 day. After one week, branching of cellular structures became evident in these developing vascular-like structures (Figure 4B). However, when the cellular structures were stained with EC-specific CD31 antibody on day 21, no expression was observed. Negative expression of CD31 was further confirmed in both 2D- and 3D-cultured hASCs and differentiated cells using Western blotting compared with positive control (HUVEC; Figure 4C). No effect of Ang-1 on expression of CD31 was detected. These results suggest that hASCs do not differentiate towards endothelial cells under described culture conditions. Therefore, we tested whether hASCs develop pericyte features instead during the culturing in EC growth medium. Western blot results showed that undifferentiated hASCs expressed alpha-smooth muscle actin (α-SMA), a late pericyte marker [35], while after differentiation, no expression was observed (Figure 4C). Further characterization using antibody against chondroitin sulfate proteoglycan/neural glial antigen 2 (NG2), an early marker for activated pericytes [36], showed higher expression in 3D-cultured hASCs and further increased expression upon EC-differentiation (Figure 4D,E).

### 3.4. Characterization of EC-Differentiated hASCs

**Figure 5:**
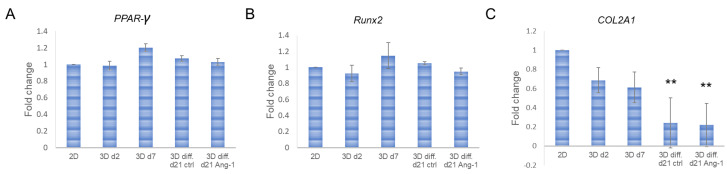
Quantitative real-time PCR of hASC-specific differentiation markers. A-C) Relative expression of adipogenic *PPAR-γ* (**A**), osteogenic *Runx2* (**B**), and chondrogenic *COL2A1* (**C**). ** *p* < 0.01 vs. 2D. N = 2–3 (3 technical replicates).

To find out the differentiation profile of hASCs in EC differentiation conditions, we further evaluated gene expressions of the cells. Expression of hASC-specific differentiation markers’ peroxisome proliferator-activated receptor gamma (*PPAR-γ*), an adipocyte specific gene, and runt-related transcription factor 2 (*Runx2*), an osteocyte-specific gene, did not significantly change during 3D culturing or differentiation compared with 2D-cultured cells (Figure 5A,B and Figure S5). On the other hand, expression of a chondrocyte-specific collagen 2A1 gene (*COL2A1*) was significantly reduced in differentiated hASCs compared with 2D control cells (Figure 5C). A slight reduction in *COL2A1* expression was also observed in undifferentiated 3D-cultured hASCs with no statistical significance.

**Figure 6:**
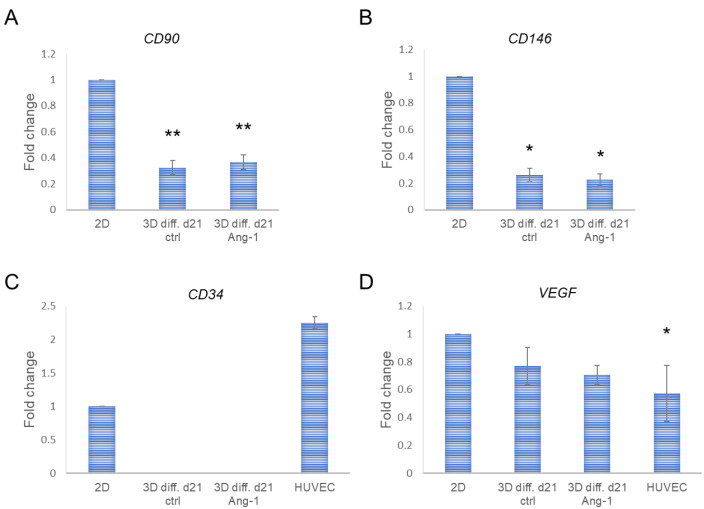
Quantitative real-time PCR of differentiated hASCs. (**A**–**C**) Relative expression of cell surface antigens *CD90* (**A**), *CD146* (**B**), and *CD34* (**C**). (**D**) Relative expression of *VEGF*. * *p* < 0.05, ** *p* < 0.01 vs. 2D. N = 2–3 (3 technical replicates).

Subsequently, we characterized 3D-cultured hASCs in EC growth medium with respect to expression of an hASC-specific cell surface antigen *CD90*, and EC-specific cell surface antigens *CD34* and *CD146*, the latter of which is a mediator of cell–cell interactions often expressed also in pericyte-like ASC populations [37,38]. Both *CD90* and *CD146* expression was significantly decreased compared with that of 2D-cultured cells (Figure 6A,B), while differentiated cells stimulated with Ang-1 did not show a difference compared with non-stimulated cells. Expression of *CD34* was not detected by qRT-PCR in hASCs cultured in EC growth medium for 21 days, while HUVECs showed high expression levels (Figure 6C). Further, we analyzed expression of *VEGF* after the differentiation process. Expression did not significantly change during culturing in EC growth medium compared with 2D control cells, in contrast to HUVEC, which showed significantly lower levels of *VEGF* (Figure 6D).

### 3.5. Angiogenic Properties of hASCs in NFC Hydrogel

**Figure 7:**
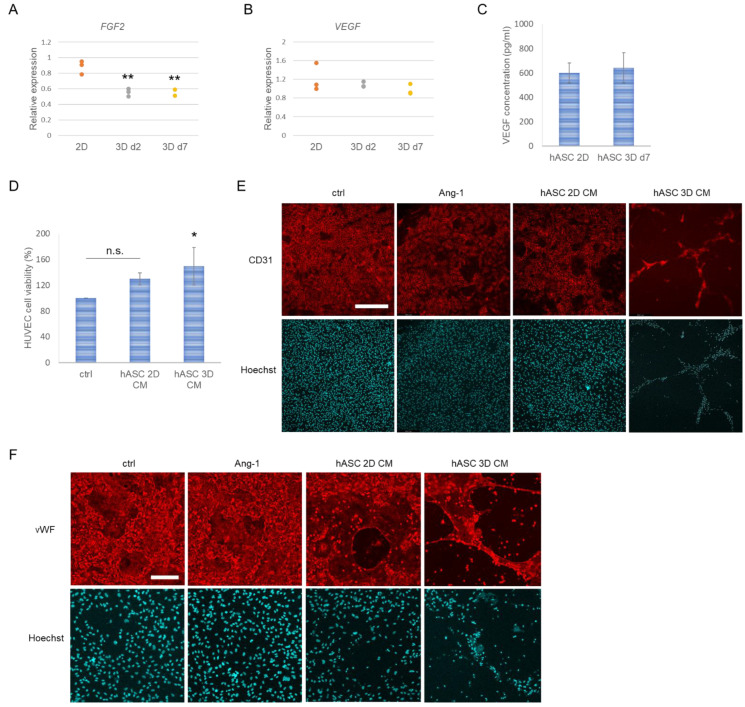
Angiogenic properties of hASCs in NFC hydrogel. (**A**) Relative expression of fibroblast growth factor 2 (*FGF2*) quantitated by qRT-PCR (*n* = 3, except for 3D d7 sample *n* = 2; 3 technical replicates). (**B**) Relative expression of vascular endothelial growth factor (*VEGF*) quantitated by qRT-PCR (*n* = 3; 3 technical replicates). (**C**) Secretion of VEGF quantitated by enzyme-linked immunosorbent assay (*n* = 3; 2 technical replicates). (**D**) Effect of hASC-conditioned medium (CM; retrieved from 2D cultures or 3D cultures in NFC hydrogel) on HUVEC cell viability (*n* = 3; 2 technical replicates). (**E**) Tube formation in HUVEC 3D cultures shown by CD31 staining of control, Ang-1, hASC 2D CM-, and hASC 3D CM-treated cells grown in 0.5% NFC hydrogel for 7 days. Scale bar, 400 µm. (**F**) HUVEC 3D cells stained with von Willebrand factor (vWF) antibody. Scale bar, 200 µm. Nuclei were stained with Hoechst. * *p* < 0.05 vs. ctrl, ** *p* < 0.01 vs. 2D. N.s., not significant.

The therapeutic potential for angiogenesis by hASCs may be improved not only via increased differentiation into ECs or other angiogenic cells but also by secretion of paracrine factors [39,40,41]. To evaluate whether hASCs retain their angiogenic characteristics in NFC hydrogel, we analyzed gene expression levels of the angiogenic growth factors fibroblast growth factor 2 (*FGF2*) and *VEGF*. Expression of *FGF2* was significantly reduced in 3D-cultured hASCs with 100 k cell density, while levels of *VEGF* were similar compared with control (Figure 7A,B). The same phenomenon was observed with 70 k cell density, which further showed decreased expression level of interleukin-6 gene (*IL-6*) but no significant change in the expression of tumor necrosis factor-alpha (*TNF-α*) inflammatory cytokine (Figure S6). We further confirmed the sustained VEGF levels by evaluating the secretion of VEGF by the cells. In 3D cultures, hASCs showed similar secretion of VEGF compared with 2D control cells (Figure 7C).

To test the paracrine effect of hASCs, we added hASC conditioned medium (CM) collected from 2D culture and 3D culture in 0.125% NFC hydrogel on HUVEC cell culture. The results showed that 3D hASC CM significantly increases the cell viability of HUVECs compared with non-treated control cells (Figure 7D). To further evaluate the effect of hASCs on endothelial cells, we cultured HUVECs in 0.5% NFC hydrogel 3D culture and treated the cells with either hASC 2D CM or hASC 3D CM or with Ang-1, which is known to improve tube formation in EC cultures [42]. The results showed that hASC 3D CM-treated HUVECs organized into tubular structures in NFC hydrogel, which was not clearly evident in control, Ang-1-treated or hASC 2D CM-treated cultures based on the observations of fluorescence imaging of CD31-stained cells (Figure 7E). 3D-cultured HUVECs also showed positive staining of vWF in every culture system (Figure 7F). In the case of hASC CM-treated cultures, tubular-like organization was observed, which was highlighted when CM was taken from 3D hASC culture. Staining of vWF showed no difference between control and Ang-1 treated cells.

## 4. Discussion

ASC-based cell therapies provide great advantages in regenerative medicine applications by accelerating healing of tissue sites and enhancing angiogenesis by natural actions without an immune response [5,8]. However, ASCs like other mesenchymal stromal cells require a proper scaffold for transplantation to protect the cells from premature degradation and for specified localization [43]. Herein we showed the possibility of using NFC hydrogel as a matrix in 3D cell culturing of hASCs. Due to the animal free-origin, NFC hydrogel would be an optimal biomaterial to be utilized in future cell-therapy applications without immunomodulatory effects. In previous studies, both neutrally charged and anionic-charged NFC hydrogel has been shown to provoke a normal inflammatory response in vivo, suggesting a safe-to-use protocol for their application in cell transplantation [44,45]. During culturing in NFC hydrogel, hASCs changed their cell spheroid formation into capillary-like structures, which could be beneficial for angiogenesis protection. In addition, hASCs typified their natural properties and showed angiogenic characteristics with potential pericyte differentiation.

One reason for the challenges involved in mesenchymal stromal cells retaining their inherent behavior after transplantation from laboratory environment to the site of action is their sensitivity to external changes [46]. Three-dimensional cell cultures in the biomimetic matrix could enable better transportation and localization of the therapeutic stromal cells. Furthermore, cells cultured in 3D could better preserve the cell viability after dosing, enabling cell–cell as well as cell–ECM interactions. This way, 3D dosing may decrease cell apoptosis caused by the loss of cell attachment, also known as anoikis-mediated death [47]. Moreover, higher cell–cell contact in 3D culture conditions might impact on higher immunosuppressive potential of the cells by released soluble factors [48,49]. In our study, we showed high viability of cells cultured in NFC hydrogel and decreased levels of IL-6. High IL-6 levels are associated with different inflammatory disease pathologies and chronic wounds [50,51]. Therefore, hASCs 3D-cultured in NFC hydrogel may show relevance in lower inflammation levels when compared to 2D culture conditions. In vivo, hASCs could reduce inflammation, e.g., by alleviating expression of inflammatory cytokines and by polarizing macrophages towards an anti-inflammatory M2-like phenotype [52], while stimulating tissue regeneration.

When the cells are cultured in a non-adherence environment, cells have the access to aggregate and form 3D spheroids, as our results proved. The spheroid formation has shown many profitable effects; for instance, enhanced properties to survive in unfavorable environments [43]. In addition, the stemness of ASCs has been shown to increase through increased cell interaction and aggregation [53], which is important for continuation of the cell lineage and for giving rise to differentiated cells. We observed that the stemness of hASCs was preserved in 3D culture when compared to the 2D control cultures. The reason for the observations of the retained and not increased stemness properties might be due to the grape-like morphology of the spheroids, which are shown to have lower cell–cell interactions when compared to more round and tighter spheroids [33]. The observed spheroid morphology followed the generally acknowledged spheroid formation process, where loose cell aggregates later form compact spheroids [54]. Most importantly, the spheroid size corresponds to the 200 µm diffusion limit found in the literature [55,56].

Spheroid formation might have affected the CD90 cell surface marker gene expression. Mesenchymal stromal cell associated cell marker CD90 is detected to be present in every ASC cell population, which was observed in our results as well. However, when hASCs were 3D cell-cultured, the expression was significantly decreased. One explanation for these differences could be the smaller cell size in the spheroids when compared with a monolayer culture. This might cause smaller detectable levels of CD90. This phenomenon was observed in the study by Santos et al. [57], where the CD90 expression was first decreased during spheroid formation and then again increased after returning to 2D conditions. Changes in the CD90 expression could be explained also by the fact that cells have differentiated into cell populations with lower CD90 expression levels. Studies have proven that when CD90 expression has been low, the osteogenic differentiation is decreased and adipogenic differentiation increased [58]. The changed expression level might be affected by the stiffness of NFC hydrogel used, which typically has much softer tissue with lower substrate elasticity than bone tissue environments. For this reason, hASCs CD90 gene expression is decreased with lower ability to differentiate into osteoblasts.

In addition to the main ASC cell markers, including CD90, CD73, and CD105, expression of CD45, CD31, CD34, and CD146 were studied. Based on the differences in these expression levels, natural ASCs have been identified into several subsets [58,59]. Four different stromal cell populations of ASCs localized around the blood vessels have been demonstrated: endothelial progenitor cells (CD45^−^, CD31^+^, CD34^+^, CD146^+^); pericytes (CD45^−^, CD31^−^, CD34^−^, CD146^+^) with the association of α-SMA^+^; supra-adventitial ASCs (CD45^−^, CD31^+^, CD34^−^, CD146^−^); and a population with characters from both of the latter two cell populations (CD146^+^, CD34^+^). Our results showed changes in the cell marker expressions during different cell culturing times and differentiation. For instance, CD34 expression is often decreased after cell culturing with further passages, and expression is affected by the culturing methods [59]. Based on our results, CD34 gene expression was highly varied between the parallel measurements in 2D conditions, which can indicate the sensitivity of this marker to culturing techniques as well as differences between donors. Being primarily a cell surface marker expressed in hematopoietic stem cells [60], CD34 expression varies in several ASC subpopulations, which can cause the variability observed in our control results. In 3D cell cultures, the gene expression of CD34 in hASCs was decreased and not detected at all after differentiation. This could mean that CD34 expression is lower during longer culturing times or that hASCs had started to differentiate, for instance into pericytes, which missed the CD34 expression. Our results further showed positive but lowered gene expression of CD146, which is associated with EC and pericyte phenotype in mesenchymal stromal cells [59].

To study possible EC formation, hASCs were cultured in EC growth medium. This would provide important support for the angiogenesis that is required for transportation of oxygen and nutrients during tissue regeneration and wound healing [61]. Cells formed vascular-like structures similar to the previously described branching structures by Klar et al. [15] during 21 days of culturing, and the formation rate was slightly enhanced when the Ang-1 stimulation was present. However, no EC-specified cell marker, CD31, expression was detected, which differs from the previous observations where endothelial formation was enhanced in 3D cell cultures [62,63]. This could suggest that hASCs would have been differentiated to other than ECs, such as pericytes. This was supported by our results of the CD34-negative and lower but maintained CD146 gene expression after differentiation. To further evaluate the possibility of hASCs differentiating into pericytes, α-SMA and NG2 expressions were determined, which have been shown to be present in mesenchymal stromal cells [64]. Interestingly, before the differentiation, α-SMA was detected but the expression was lacking after the differentiation. Decrease in α-SMA expression was also observed in the study by Talele et al. [65] where the expression levels in the stromal cells were in correlation with the substrate properties. When α-SMA-positive cells were cultured in a soft substrate environment, the expression level was decreased and finally disappeared. Expression of NG2, on the other hand, was most prominently observed in the differentiated 3D-cultured hASCs. These findings are supported by a previous study by Mazzocchi et al. [66], who showed increased NG2 expression after culturing of ASCs in EC differentiation medium compared with culturing in proliferation medium. This would suggest that instead of EC formation, hASCs would be more likely to differentiate into pericyte-like cell populations in EC medium. However, the used cell culture conditions with low fiber concentration of NFC hydrogel decreased the appearance of the pericyte-specific cell markers. In the future, it would be worthwhile to study whether higher NFC fiber concentration would support endothelial differentiation of hASCs.

Although no ECs were proven to be present in these culture conditions, faster tube formation was observed in our results when the hASC cultures were stimulated with Ang-1. Ang-1 is known to stabilize chemotaxis and increase tube formation in EC cultures [67]. The effects of Ang-1 in promoting the organization of the cells into tube formation are primarily based on the Tie2-receptors in ECs [68]. It might be that although stimulation did not enhance endothelial properties, Ang-1 has the ability to affect other cell types than ECs. For instance, Tie2receptors have been found in pericytes, in which the Ang-1 increases cell survival and migration [69]. Furthermore, Ang-1 stimulation has proved protectivity for mesenchymal stromal cells after transplantation [70], which could increase the effectiveness of stromal cell-based therapies.

Differentiation of hASCs was further studied by measuring specific gene expressions associated with adipocytes (PPAR-γ), osteocytes (Runx2), or chondrocytes (COL2A1). While PPAR-γ and Runx2 gene expressions did not show any significant difference between 2D and 3D cell cultures, the COL2A1 gene expression was significantly reduced, suggesting a decreased potential of cells to differentiate towards chondrocytes but not towards adipocytes or osteocytes. This could be associated with the results from the paracrine analyses, where the angiogenic growth factor *FGF2*, which is proven to have an important role in chondrogenesis [71], showed decreased expression in 3D cell culture. FGF2 plays a role in the EC formation and its absence has decreased the expression levels of EC-specified markers, such as CD31 [72]. Therefore, the decrease in FGF2 levels could have resulted in the undifferentiation of hASCs into ECs observed in this study.

One of the most important features of hASCs showing tissue regeneration properties is based on their paracrine factors [13]. According to our results, hASCs retained their paracrine secretion in 3D cell culture, which was evaluated with respect to VEGF expression and secretion levels. The paracrine properties of 3D-cultured hASCs were demonstrated by increased viability of HUVEC cells in 2D culture and strong tubular-like formation in 3D culture after treating them with hASC-conditioned medium. In the case of hASC 2D-conditioned medium, no significant increase in HUVEC viability was observed when compared to the control, and only slight effects on organization of HUVECs was shown with vWF staining. These findings highlighted again the importance of 3D culture conditions when considering angiogenesis protection by ASC-cell based therapies. The exact paracrine mechanism affecting HUVEC tube formation by hASCs, whether it be a secretory molecule or occurs via extracellular vesicle (EV) secretion, was not evaluated in this study and should be analyzed in more detail in the future. In a previous study by Cinnici et al. [73], MSC-secreted EVs were studied and found to be a crucial part of releasing proangiogenic molecules, leading to increased vascularization. In addition to the culturing conditions, these paracrine properties can be affected by the transplantation mechanisms and better results have been shown with the local administration of mesenchymal stromal cells instead of systemic exposure [74]. NFC hydrogel would be an optimal scaffold in this sense since it can be administered locally at the injury site and protect the stromal cells from premature degradation. Furthermore, this biomimetic material could enable stable release of paracrine factors and control the location of the stromal cells.

The observed cell marker gene/protein expression of the 3D cell-cultured hASCs were CD31^−^, CD34^low^ (before differentiation)/CD34^−^ (after differentiation), CD146^low^, and CD166 with remained pluripotency cell marker expression levels and decreased CD90 expression. Cells showed α-SMA expression in the undifferentiated cells and more prominent NG2 expression after differentiation. Gene expression of VEGF was preserved. When the expression profile was compared with the cell markers present in pericytes, similarities could be observed but with lower expression levels. This could be an effect of the used culture material’s properties: as the used 0.125% NFC hydrogel represents a soft tissue substrate with viscous-fluid-like behavior [75] based on our rheological measurements, it might have affected the observed differentiation preferences of hASCs. It could provide better conditions for adipogenic differentiation, as we observed a slight increase of PPAR-γ gene expression in 3D-cultured cells on day 7, which, however, was not statistically significant.

Effects of scaffold thickness on cell behavior and differentiation have previously been studied. Lou et al. [76] showed that human pluripotent stem cells changed their spheroid formation behavior when different fiber concentrations of NFC hydrogel were applied. Regarding endothelial differentiation, studies have shown differing differentiation potential of mesenchymal stromal cells depending on the scaffold stiffness [77,78,79]. With respect to stiffness control, NFC hydrogel could become a highly beneficial material since it has a fiber concentration directly proportional to the mechanical properties [25] that can easily be adjusted when needed by diluting the hydrogel or concentrating the dried form of NFC, as explained in our previous studies [80]. This study proved NFC hydrogel to preserve the natural hASCs properties and their viability with the ability to change the morphology from 3D cell spheroids into vascular-like structures during culturing.

## 5. Conclusions

Donor hASCs were successfully 3D cell-cultured in NFC hydrogel with high cell viability. Instead of differentiation into ECs, hASCs showed angiogenic potential by pericyte-like differentiation and paracrine characteristics based on the observed cell marker expressions. Differentiation properties of hASCs might have been affected by the soft tissue-referred elastic stiffness of NFC hydrogel. Based on this study, NFC hydrogel is a suitable matrix for culturing hASCs with angiogenic properties, and a potential scaffold to protect the cells from premature degradation and for more specific targeting after transplantation to the injury site.

## Figures and Tables

**Table 1:** Primer sequences used in quantitative real-time PCR assays.

Gene	Forward Primer	Reverse Primer
***β-2-m***	CTCGCGCTACTCTCTCTTTCTG	GCTTACATGTCTCGATCCCACT
***CCND1***	TATTGCGCTGCTACCGTTGA	CCAATAGCAGCAAACAATGTGAAA
***CD34***	TCTGGATCAAAGTAGGCAGGA	GATCCAGCCTCAGAGGAAGA
***CD73***	CTTAACGTGGGAGTGGAACC	TCTAGCTGCCATTTGCACAC
***CD90***	CGCTCTCCTGCTAACAGTCTT	CAGGCTGAACTCGTACTGGA
***CD105***	CTAACTGGCAGGGGAGACAG	CTCCATGTGGCAGGAGCTA
***CD146***	ATCGCTGCTGAGTGAACCACAG	CTACTCTCTGCCTCACAGGTCA
***CD166***	ATTGAAGTTTTATTTGGCAGGAA	GGCTTAGCCATGCAAAACA
***COL2A1***	CGTCCAGATGACCTTCCTACG	TGAGCAGGGCCTTCTTGAG
***FGF2***	ATGGCAGCCGGGAGCATCACCCACG	TCAGCTCTTCGCAGACATTGGAAG
***IL-6***	AACCTGAACCTTCCAAAGATGG	TCTGGCTTGTTCCTCACTACT
***NANOG***	GCAGAAGGCCTCAGCACCTA	GGTTCCCAGTCGGGTTCAC
***OCT4***	CAGTGCCCGAAACCCACAC	GGAGACCCAGCAGCCTCAAA
***PPAR*** ***γ***	TCAGCGGGAAGGACTTTATGTATG	TCAGGTTTGGGCGGATGC
***Runx2***	GTCTTACCCCTCCTACCTGA	TGCCTGGCTCTTCTTACTGA
***SOX2***	CTCCGGGACATGATCAGC	GGTAGTGCTGGGACATGTGAA
***TNF-α***	ATGAGCACTGAAAGCATGATCC	GAGGGCTGATTAGAGAGAGGTC
***VEGF***	TGCTTCTGAGTTGCCCAGGA	TGGTTTCAATGGTGTGAGGACATAG

## Data Availability

The data presented in this study are available on request to the corresponding author.

## Supplementary Materials

The following supporting information can be downloaded at: https://www.mdpi.com/article/10.3390/biomedicines10102584/s1, Table S1: Details of antibodies applied for human ASC cell surface marker analyses by flow cytometry; Table S2: Results of human ASC cell surface marker analysis by flow cytometry; Figure S1: Dot plots of human ASC cell surface marker analyses by flow cytometry; Figure S2: Cell viability of hASCs in different NFC concentrations with different cell densities; Figure S3: Rheological characterization of NFC hydrogel; Figure S4: Quantitative real-time PCR of specific cell surface markers, pluripotency markers, and a cell cycle marker in hASCs cultured in 0.125% NFC hydrogel with 70 k cell density on d7 compared with 2D-cultured control cells; Figure S5: Quantitative real-time PCR of hASC-specific differentiation markers in 0.125% NFC hydrogel with 70 k cell density on d7 compared with 2D-cultured control cells; Figure S6: Relative expression of angiogenic growth factors.
