# Supplementary material for: Angiogenic Potential of Human Adipose-Derived Mesenchymal Stromal Cells in Nanofibrillated Cellulose Hydrogel
Source: Biomedicines. 2022 Oct 15;10(10):2584. doi: 10.3390/biomedicines10102584 (PMC9599553; doi:10.3390/biomedicines10102584)
Supplement: Supplementary file 1 [file biomedicines-10-02584-s001.zip › biomedicines-1835140-supplementary.pdf]

**Supporting information**

# Angiogenic potential of human adipose derived mesenchymal stromal cells in nanofibrillated cellulose hydrogel

Elle Koivunotko [1], Jasmi Snirvi [1], Arto Merivaara [1], Riina Harjumäki [1], Swarna Rautiainen [1], Minna Kelloniemi [2], Kirsi Kuismanen [3], Susanna Miettinen [4,5], Marjo Yliperttula [1,*] and Raili Koivuniemi [1,*]

[1] Division of Pharmaceutical Biosciences, Drug Research Program, Faculty of Pharmacy, University of Helsinki, 00790 Helsinki, Finland
[2] Department of Plastic and Reconstructive Surgery, Tampere University Hospital, 33520 Tampere, Finland
[3] Department of Obstetrics and Gynecology, Tampere University Hospital, 33520 Tampere, Finland
[4] Faculty of Medicine and Health Technologies, University of Tampere, 33520 Tampere, Finland
[5] Research, Development and Innovation Centre, Tampere University Hospital, 33520 Tampere, Finland
* Correspondence: marjo.yliperttula@helsinki.fi (M.Y.); raili.koivuniemi@helsinki.fi (R.K.)

## Supplemental materials and methods

### Rheological measurements

Choosing of the right fiber concentration of nanofibrillated cellulose (NFC) hydrogel for the cell experiments was based on the Young's modulus measurements, from which the hydrogel with the stiffness that resembles the most the extracellular matrix of the adipose stem cells was chosen [1]. Young's modulus measurements were implemented in collaboration with UPM Biomedicals, Finland and were following the protocol introduced in Bhattacharya et al. (2012) [2]. Measurements were done for NFC hydrogels with concentrations between 0.1% – 0.9% using a stress controlled rotational rheometer (AR-G2, TA instruments, UK).

HAAKE Viscotester iQ Rheometer (Thermo Fisher Scientific, Karlsruhe, Germany) was used for shear loss modulus (G``) and shear storage modulus (G`) measurements of NFC hydrogel (GrowDex®; UPM Biomedicals, Finland) with 0.125% (m/V) fiber content. Measurements were implemented at controlled temperature (+25°C) and double cap geometry was used with parallel 25 mm diameter steel. Based on the viscoelastic region obtained from the measured constant amplitude sweep (constant angular frequency $\omega = 1$ Hz and oscillatory stress between $1 \times 10^{-4}$–500 Pa), the oscillatory stress was chosen ($\tau = 0.5$ Pa). The angular frequency ranged from 0.6 to 18.5

rads$^{-1}$. Measurements were implemented in triplicate and analyzed from 24 time points with HAAKE RheoWin 4.0 software (Thermo Fisher Scientific).

**Supplemental Table S1**. Details of antibodies applied for human ASC cell surface marker analyses by flow cytometry.

| **Antibody** | **Clone** | **Manufacturer** | **Amount (µl)** |
|---|---|---|---|
| PE-Cy7 Mouse Anti-Human CD14 | M5E2 | BD pharmingen | 2 |
| PE-Cy7 Mouse Anti-Human CD19 | SJ25C1 | BD pharmingen | 4 |
| anti-human Cd34 APC-conjugated | 4H11[APG] | Immunotools | 10 |
| CD45RO APC | UCHL-1 | BD pharmingen | 3 |
| CD49d Mouse Anti-Human PE | 9F10 | BD Pharmingen | 10 |
| PE Conjugated Mouse Anti-human CD73 | AD2 | BD pharmingen | 10 |
| APC Mouse Anti-Human CD90 | 5E10 | BD pharmingen | 0.3 |
| Anti-human CD105/Endoglin-Phycoerythrin | 166707 | R&D systems | 10 |
| PE-Cy5 Mouse Anti-Human CD106 | 51-10C9 | BD Pharmingen | 10 |
| anti-human HLA-ABC PE-conjugated | W6/32 | Immnotools | 10 |
| anti-human HLA-DR PE-conjugated | MEM-12 | Immnotools | 10 |
| pe Mouse Anti-Human CD3 | UCHT1 | BD pharmingen | 10 |
| Anti-human Integrin αL/CD11a-Allophycocyanin | 345913 | R&D systems | 10 |
| ICAM-1/CD54-Fluorecein | BBIG-l1 | BD pharmingen | 10 |
| | | BD pharmingen | 10 |
| Anti-human B7-1/CD80-Phycoerythrin | 37711 | R&D systems | 10 |
| Anti-human B7-2 (CD86)-Phycoerythrin | 37301 | R&D systems | 10 |
| PE-Cy™7 Mouse Anti-Human CD11b | ICRF44 | BD pharmingen | 5 |

## Supplemental Results

**Supplemental Table S2.** Results of human ASC cell surface marker analysis by flow cytometry.

| Antigen | Surface protein | Mean | SD |
|---|---|---|---|
| CD3* | T-cell signal transduction | 0.6 | 0.3 |
| CD11a/b* | Cell interactions and T-cell mediated killing | 14.4 | 21.5 |
| CD14 | Bacterial lipopolysaccharide binding protein | 3.8 | 2.9 |
| CD19 | B-lymphocyte-lineage differentiation antigen | 3.2 | 2.9 |
| CD34 | Sialomucin-like adhesion molecule | 23.5 | 14.9 |
| CD45 | Leukocyte common antigen | 5.9 | 5.0 |
| CD54* | Cell adhesion, lymphocyte activation and migration | 21.7 | 16.8 |
| CD73 | Ecto-5'-nucleotidase | 94.9 | 6.2 |
| CD80* | Lymphocyte activation | 2.9 | 4.2 |
| CD86* | Regulates T-cell activation | 3.2 | 3.7 |
| CD90 | Thy-1 (T-cell surface glycoprotein) | 97.7 | 3.0 |
| CD105 | SH-2 endoglin | 99.6 | 0.4 |
| HLA-DR | Major histocompatibility class II antigens | 2.7 | 3.0 |

n = 9; *n = 7

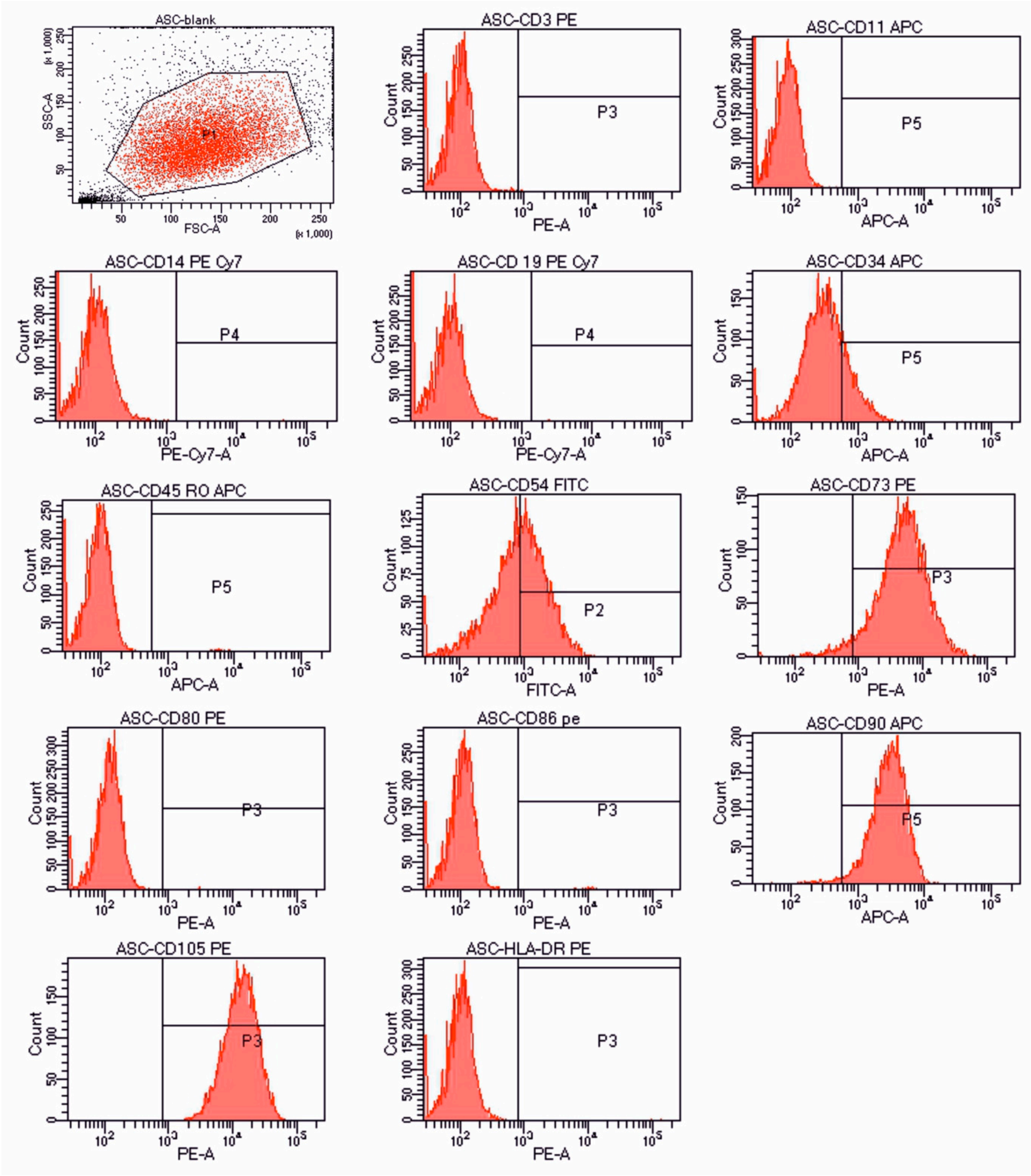


**Supplemental Figure S1.** Dot plots of human ASC cell surface marker analyses by flow cytometry.

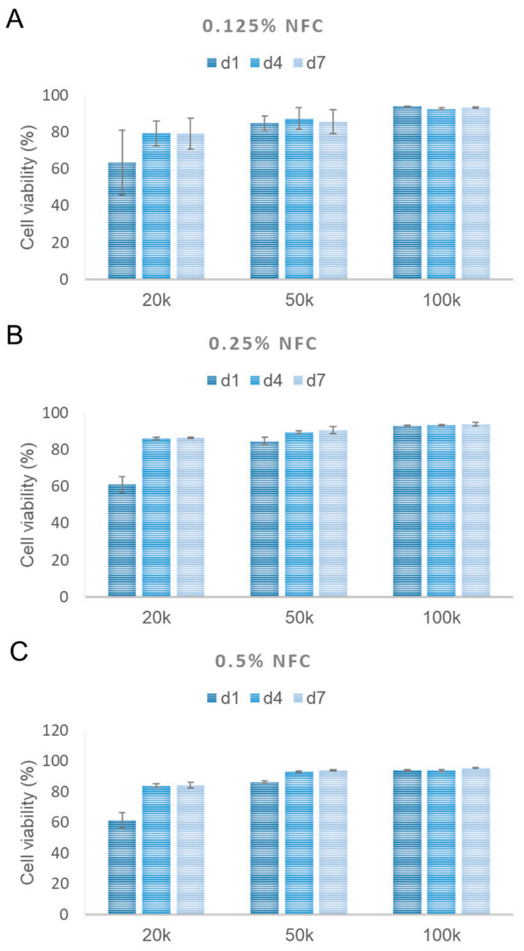


**Supplemental Figure S2.** Cell viability of hASCs in different NFC concentrations with different cell densities. A) 0.125%, B) 0.25%, and C) 0.5% NFC hydrogel.

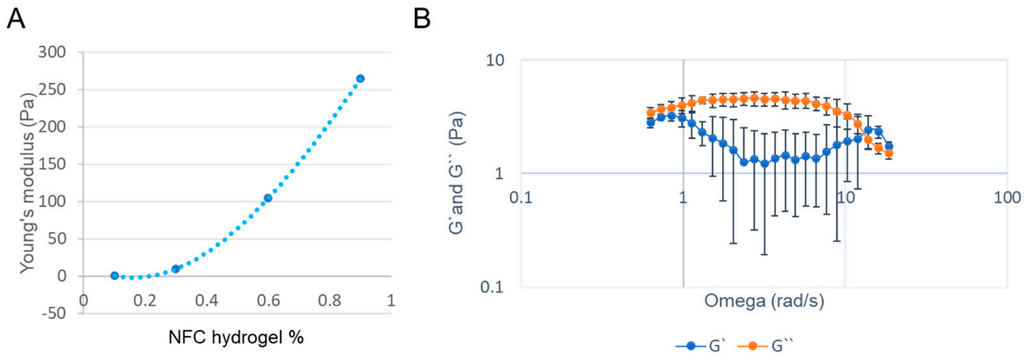


**Supplemental Figure S3.** Rheological characterization of NFC hydrogel. A) Young's moduli of different NFC hydrogel concentrations. B) Storage (G') and loss modulus (G'') of 0.125% NFC hydrogel.

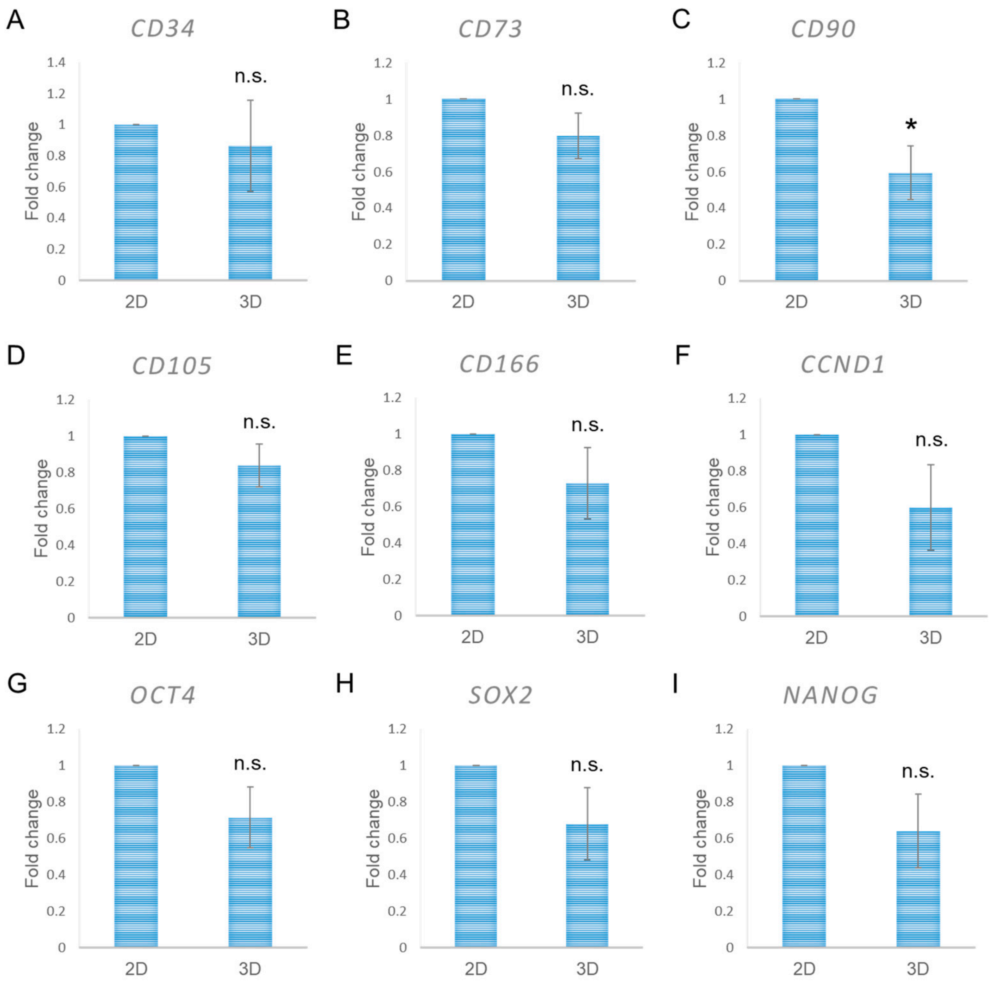


**Supplemental Figure S4.** Quantitative real-time PCR of specific cell surface markers, pluripotency markers and a cell cycle marker in hASCs cultured in 0.125% NFC hydrogel with 70k cell density on d7 compared with 2D cultured control cells. A-D) Relative expression of ASC specific cell surface markers *CD34* (A), *CD73* (B), *CD90* (C), *CD105* (D) and *CD166* (E). F) Relative expression of a cell cycle activator *CCND1*. G-I) Relative expression of pluripotency markers *OCT4* (E), *SOX2* (F) and *NANOG* (G). * $p < 0.05$. N.s., not significant.

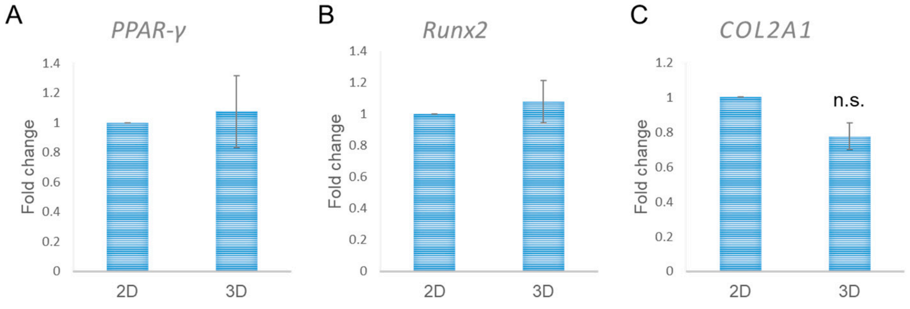


**Supplemental Figure S5.** Quantitative real-time PCR of hASC specific differentiation markers in 0.125% NFC hydrogel with 70k cell density on d7 compared with 2D cultured control cells. Relative expression of adipogenic *PPAR-γ* (A), osteogenic *Runx2* (B) and chondrogenic *COL2A1* (C). N.s., not significant.

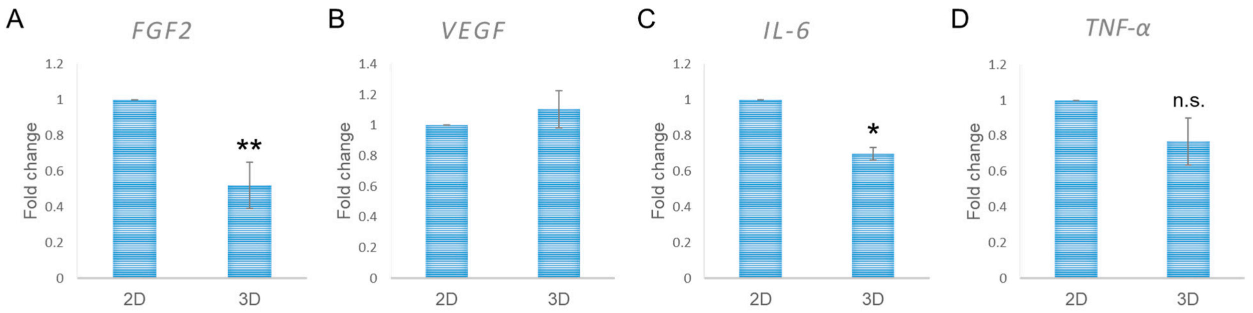


**Supplemental Figure S6.** Relative expression of angiogenic growth factors *FGF2* (A) and *VEGF* (B), and cytokines interleukin-6 (*IL-6*) (C) and tumor necrosis factor-α (*TNF-α*) (D) analyzed by qRT-PCR in hASCs cultured in 0.125% NFC hydrogel with 70k cell density on d7 compared with 2D cultured control cells. * $p < 0.05$, ** $p < 0.01$. N.s., not significant.

## Supplemental references

1. Kerstyn C, Fleck NA. A micromechanical model for the Young's modulus of adipose tissue. International Journal of Solids and Structures 47, 2010:21. https://doi.org/10.1016/j.ijsolstr.2010.07.001.

2. Bhattacharya M, Malinen MM, Lauren P, Lou YR, Kuisma SW, Kanninen L, et al. Nanofibrillar cellulose hydrogel promotes three-dimensional liver cell culture. Journal of Controlled Release, 2012:164. https://doi.org/10.1016/j.jconrel.2012.06.039.
